# Designing Crystalline/Amorphous NVNPF/NCK Cathode Toward High‐Performance Fully‐Printed Flexible Aqueous Rechargeable Sodium‐Ion Batteries (ARSIBs)

**DOI:** 10.1002/advs.202416120

**Published:** 2025-02-04

**Authors:** Hehe Ren, Jing Liang, Qun Liu, Yuanjie Wei, Wei Wu

**Affiliations:** ^1^ Laboratory of Printable Functional Materials and Printed Electronics School of Physics and Technology Wuhan University Wuhan 430072 P. R. China

**Keywords:** battery‐supercapacitor composite materials, crystalline and amorphous phases, fully‐printed flexible ARSIBs, high performance, Ni^2+^ doping

## Abstract

The flexible ARSIBs have great potential in portable and wearable electronics due to their high cost‐effectiveness, safety, and amazing flexibility. Nevertheless, achieving both outstanding flexibility and high energy density remains a challenge. Herein, a battery‐supercapacitor composite material Na_3_V_1.95_Ni_0.05_(PO_4_)_2_F_3_/10%NC‐KOH (NVNPF/NCK) with coexistence of crystalline and amorphous phases is fabricated by loading nitrogenous carbon (NC) onto Na_3_V_1.95_Ni_0.05_(PO_4_)_2_F_3_ (NVNPF) and etching with KOH. It demonstrates high specific capacity (187.26 mAh g^−1^), ultrahigh energy density (262.16 Wh kg^−1^), and excellent cycle performance (the capacity retention is 81% at 1 C after 500 cycles). The high performance is achieved by doping Ni^2^⁺ loading NC and etching with KOH, which generates vacancy defects, enhances structural stability, and accelerates ion‐diffusion kinetics. Furthermore, the fully‐printed ARSIBs (F‐NTP//NVNPF/NCK) with high specific capacity (60.37 mAh g^−1^), amazing energy density (72.44 Wh kg^−1^), and excellent cycle performance, are fabricated using screen‐printing technique based on the NVNPF/NCK cathode and NaTi_1.7_Fe_0.3_(PO_4_)_3_ (F‐NTP) anode. To the best of the authors' knowledge, F‐NTP//NVNPF/NCK is the highest‐performing fully‐printed flexible ARSIB to date. In particular, these batteries can achieve tunability in shape and size, integration, and high‐throughput manufacturing. Thus, this work can offer greater possibilities for the development of high‐performance flexible ARSIBs.

## Graphical abstract

The battery‐supercapacitor composites Na_3_V_1.95_Ni_0.05_(PO_4_)_2_F_3_/10%NC‐KOH (NVNPF/NCK) with the coexistence of crystalline and amorphous phases are fabricated by doping Ni^2+^, loading nitrogenous carbon (NC), and etching with KOH. The F‐NTP//NVNPF/NCK batteries with high energy density, impressive cycle performance, and excellent mechanical performance are prepared by the screen‐printing technique, which are fully‐printed flexible ARSIBs with the highest performance reported so far.

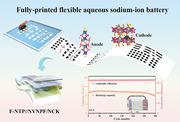

## 1 Introduction

Emerging wearable flexible electronic devices, which are flexible, lightweight, and miniaturized, have promoted the development of flexible batteries that prioritize safety, environmental sustainability, and cost‐effectiveness.^[^ ^1^, ^2^, ^3^ ^]^ Flexible ARSIBs are similar in principle to lithium‐ion batteries (LIBs), with abundant sodium resources and environmentally‐friendly aqueous electrolytes. These advantages make them promising to meet the requirements of portable and wearable electronics for low cost, excellent flexibility, and high safety, attracting increasing attention.^[^ ^4^, ^5^, ^6^ ^]^ Although conventional LIBs have been commercialized, they still face challenges in high flexibility, sustainability, and wearable applications due to strict manufacturing processes, limited lithium resources, and flammable organic electrolytes.^[^ ^7^, ^8^, ^9^ ^]^ In recent years, sodium superionic conductor‐type Na_3_V_2_(PO_4_)_3_ (NVPF) has a stable framework structure and 3D network of interconnected channels, exhibiting a high theoretical capacity and making it a promising cathode material for ARSIBs.^[^ ^10^, ^11^ ^]^ However, the large radius of Na^+^ and the instability of NVPF materials in aqueous electrolytes, result in slow diffusion kinetics and unsatisfactory cyclic stability, and NVPF can only present 100 cycles as the cathode for ARSIBs.^[^ ^12^, ^13^ ^]^ Therefore, it is of great significance to explore effective approaches to optimize the performance of active materials.

Recently, various optimization strategies such as carbon coating, composite modification, heteroatom doping, and crystal growth control have been reported to obtain active materials with excellent electrochemical performance and appealing stability.^[^ ^14^, ^15^ ^]^ The synergistic action of heteroatom doping and crystal growth control can effectively solve the issues of low intrinsic electronic conductivity and instability of NVPF. Replacing V elements with affordable and environmentally friendly transition metals (such as Mn, Cr, Fe, Zr, Ti, etc.) can improve conductivity, increase vacancy defects, and accelerate ion‐diffusion kinetics.^[^ ^16^, ^17^, ^18^, ^19^, ^20^ ^]^ Moreover, the nitrogenous carbon (NC) loading and acid/alkali etching methods can promote the growth of the crystal material toward a disordered structure and obtain the high‐performance active material with the coexistence of crystalline and amorphous phases. In summary, abundant surface and internal defects in the amorphous structure can effectively decrease the volume expansion caused by the insertion/extraction of Na^+^, and a stable crystalline structure can maintain the structural stability of materials.^[^ ^21^, ^22^ ^]^

According to previous reports, most flexible sodium‐ion batteries are typically pouch‐type cells with nonplanar stacked geometries, which are bulky, limited in flexibility, and not suitable for large‐scale manufacturing in practical applications. Among the various manufacturing methods for flexible batteries, we utilized the screen‐printing technique to manufacture a variety of fully‐printed flexible devices based on our previous research advancements.^[^ ^23^, ^24^ ^]^ Screen‐printing technique offers a facile manufacturing process, low cost, and can ensure the flatness and consistency of printed electrode surface.^[^ ^25^, ^26^, ^27^ ^]^ In addition, according to the specific design requirements of wearable electronics, screen‐printed devices can achieve the controllability of thickness, size, shape, and composition, are compatible with serial and parallel integration, and can be manufactured in high throughput.

In this paper, we fabricate a series of novel composites by doping Ni^2+^, loading NC and etching with KOH onto NVPF, and applied as the cathode of ARSIBs. The reasonable doping ratio of Ni^2+^ and a suitable amount of NC and KOH are explored to get the high‐performance cathode Na_3_V_1.95_Ni_0.05_(PO_4_)_2_F_3_/10%NC‐KOH (NVNPF/NCK), which is a battery‐supercapacitor composite with the coexistence of crystalline and amorphous phases. Moreover, inspired by the high‐performance NVNPF/NCK and NaTi_1.7_Fe_0.3_(PO_4_)_3_ (F‐NTP) electrode materials, fully‐printed flexible ARSIBs (F‐NTP//NVNPF/NCK) are prepared by the screen‐printing technique. The batteries show excellent electrochemical performance, high flexibility, and appealing stability under extreme conditions, including bending, puncturing, low temperature, cutting, and washing. According to the design requirements of wearable electronics, this type of flexible battery shows tunability in thickness, shape, and size, and exhibits compatible series and parallel modularization, making it promising in high‐throughput manufacturing. To the best of our knowledge, NVNPF/NCK as a cathode of ARSIBs shows the highest performance among many V‐based polyanionic compounds, and F‐NTP//NVNPF/NCK is the highest performing fully‐printed flexible ARSIB as yet. Consequently, these fully‐printed flexible ARSIBs can meet the requirements for high cost‐effectiveness, excellent flexibility, and scalable integration, which provide a promising pathway for the advancement of flexible electronics.

## 2 Results and Discussion

### 2.1 Morphologies and Structural Characterizations of NVNPF/NCK Composites

**Figure 1:**
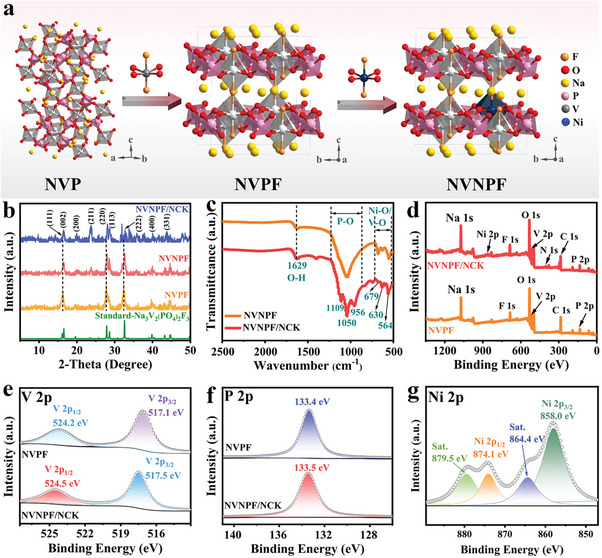
a) The variations of crystal structure. b) XRD patterns. c) FTIR spectrum. The XPS spectra of d) full spectrum, e) V 2p for NVPF and NVNPF/NCK, f) P 2p for NVPF and NVNPF/NCK, and g) Ni 2p for NVNPF/NCK.

The monoclinic phase NVP with a 3D open framework is composed of sharing corners between VO_6_ octahedra and PO_4_ tetrahedra.^[^ ^28^, ^29^, ^30^ ^]^ The doping of F^−^ in NVP further improves the stability of NVP material, and forms V─F bond with V^3+^, which causes crystal phase transformation and forms a stable tetragonal phase NVPF. **Figure** **1**a displays the unit cell structure of tetragonal phase NVPF and Ni‐doped Na_3_V_1.95_Ni_0.05_(PO_4_)_2_F_3_ (NVNPF), the [M_2_(PO_4_)_2_F_3_]_3_^−^ (M = V, Ni) structural unit is made up of sharing corners with a M_2_O_8_F_3_ (M = V, Ni) octahedron and three PO_4_ tetrahedra in NVPF and NVNPF frameworks. Moreover, these units are arranged into [M_2_(PO_4_)_2_F_3_]_∞_ bands, and the adjacent bands are connected by the PO_4_ tetrahedron, which can create a 3D open framework with large gap spaces and interconnected 3D network conduction pathways that greatly facilitate Na^+^ transport.^[^ ^31^, ^32^ ^]^ In Figure S1a (Supporting Information), X‐ray diffraction (XRD) patterns of Na_3_V_2‐2x_Ni_2x_(PO_4_)_2_F_3_ (NVNPF x%, *x* = 0, 2.5, 5, 10, 20) samples show diffraction peaks characteristic of the tetragonal phase,^[^ ^33^ ^]^ without impurity peaks, indicating that Ni^2+^‐doping has no impact on the crystal structure. Since the smaller ionic radius of Ni^2+^ compared to V^3+^, and Ni─O and Ni─F bond lengths are shorter than V‐O and V‐F, resulting in slight lattice contraction of NVNPF, and the XRD characteristic peaks of NVNPF slightly shift to the right (Figure 1b).^[^ ^34^ ^]^ The doping of Ni^2+^ promotes the lattice contraction of NVNPF, which shortens the Na^+^ diffusion distance, improving the material stability.

To obtain high‐performance materials with excellent cycling stability, the NVNPF is combined with NC and etched by KOH to fabricated Na_3_V_1.95_Ni_0.05_(PO_4_)_2_F_3_/n%NC‐KOH composites (NVNPF/NCK n%, *n* = 0, 5, 10, 20). The XRD characteristic peaks of the NVNPF/NCK n% composites in Figure S1b (Supporting Information) are in agreement with the standard cif file of tetragonal phase NVPF. Moreover, with the increasing addition of NC and KOH, the strength of the characteristic peak decreased significantly, which indicates the crystallinity of materials decreased, and the NVNPF/NCK n% composites exhibit both crystalline and amorphous structure. Additionally, the surface morphology and structural composition of NVNPF and NVNPF/NCK are examined by scanning electron microscopy and energy dispersive spectroscopy. As shown in Figure S2 (Supporting Information), NVNPF shows a heavily aggregated irregular block structure with abundant pores on the surface. In Figure S3 (Supporting Information), NVNPF is combined with NC and etched with KOH to obtain NVNPF/NCK composites with abundant active sites and large specific surface area (SSA). Compared to NVNPF, the surface of composite materials is relatively rough, which proves the successful load of NC on the NVNPF surface. The N_2_ isothermal adsorption‐desorption experiment is tested to evaluate the SSA and pore size distribution of NVNPF and NVNPF/NCK (Figure S4, Supporting Information). The NVNPF/NCK shows a significantly larger SSA (27 m^2^ g^−1^) compared to NVNPF (6 m^2^ g^−1^). Furthermore, the pore‐size distribution (inset) further confirms that both NVNPF and NVNPF/NCK are mesoporous materials, with NVNPF/NCK displaying a more abundant pore structure. These results suggest that NVNPF/NCK with the larger SSA, can provide more extensive contact with the electrolyte, facilitating the diffusion of Na⁺.

As shown in Figure 1c, the Fourier‐transform infrared spectroscopy (FTIR) is measured to further analyze the composition of the materials. The signal peak of O─H bonds is located at 1629 cm^−1^,^[^ ^35^ ^]^ while the asymmetric stretching vibrations of the P─O bond are responsible for the signals at 956, 1050, and 1109 cm^−1^.^[^ ^36^ ^]^ The signals at 564, 630, and 679 cm^−1^ are associated with V─O/Ni─O bonds,^[^ ^37^, ^38^ ^]^ confirming the successful synthesis of NVNPF and NVNPF/NCK materials without any impurities. In addition, the structure composition of the NVNPF/NCK composites is further investigated by Raman spectroscopy (Figure S5a, Supporting Information). Additionally, the Raman spectroscopy is used to further analyze the structure of NVNPF/NCK composites, the D band and G band are located at 1300 and 1600 cm^−1^, which represents disordered carbon and graphite carbon, respectively.^[^ ^39^ ^]^ For NVNPF and NVNPF/NCK, the intensity ratios of D band to G band (I_D_/I_G_) are 0.91 and 0.94, respectively, indicating that the NC loading and KOH etching onto NVNPF can increase surface defects and decrease the crystallinity of materials.

The X‐ray photoelectron spectroscopy (XPS) is measured to evaluate the elemental composition of NVPF and NVNPF/NCK. As shown in Figure 1d, the elements Na, Ni, F, O, V, N, C, and P elements are presented in the full XPS spectrum of NVNPF/NCK, further demonstrating the successful synthesis of the NVNPF/NCK composites. Figure 1e shows the V 2p spectrum of NVPF and NVNPF/NCK, the V 2p_1/2_ and V 2p_3/2_ states of V^3+^ are linked to the signal peaks at 524.2, 524.5 eV and 517.1, 517.5 eV, respectively,^[^ ^40^ ^]^ suggesting that Ni^2+^‐doping has no influence on the valence state of V ions. The signals of P 2p for NVPF and NVNPF/NCK are located at 133.4 and 133.5 eV, respectively, which corresponds to the PO_4_ units (Figure 1f).^[^ ^41^ ^]^ Moreover, the F 1s XPS spectrum is displayed in Figure S5b (Supporting Information); the V─F bond is represented by the signals at 684.6 and 684.9 eV, and the binding energy at 686.7 eV in the NVNPF/NCK samples corresponds to C─F bond, indicating the NC is successfully loaded on the NVNPF.^[^ ^42^ ^]^ In Figure 1g, two peaks at 864.4 and 879.5 eV reflect the satellite peaks and the peaks at 879.5 and 858.0 eV are the signals of Ni 2p_1/2_ and Ni 2p_3/2_, proving the existence of Ni^2+^.^[^ ^43^ ^]^ The above XPS results demonstrate the successful doping of Ni^2+^ and a combination of NVNPF and NC, without effect on the valence states of other elements.

### 2.2 Electrochemical Performance of NVNPF/NCK Cathode

**Figure 2:**
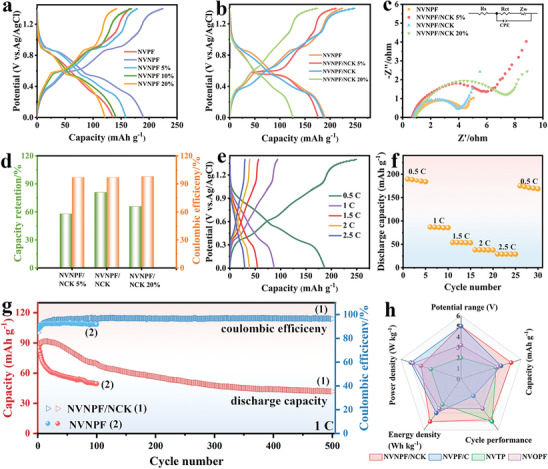
The charge/discharge curves of a) NVNPF x% and b) NVNP/NCK n% samples. c) EIS results. d) Cycle performance. e) The GCD curves and f) the rate capability of NVNPF/NCK. g) Cycle performance. h) Radar chart comparing the electrochemical performances of NVNPF/NCK with reported V‐based polyanionic compounds.

Based on our previous research,^[^ ^13^ ^]^ the 17 mol kg^−1^ NaClO_4_/ethylene glycol (17 m NaClO_4_‐EG) electrolyte is prepared with 17 m NaClO_4_ “water‐in‐salt” electrolyte and EG co‐solvent, which can not only inhibit the side reactions of hydrogen evolution and oxygen evolution but also form new hydrogen bonds between EG and H_2_O molecules to obtain a low‐freezing‐point electrolyte. As shown in Figure S9a (Supporting Information), to explore the effect of different Ni^2+^‐doping ratios on NVPF materials, the cyclic voltammetry (CV) results (1 mV s^−1^) are measured at 0–1.4 V in 17 m NaClO_4_‐EG electrolyte. Due to the V^3+^/V^4+^ redox couple and the Na^+^ insertion/extraction behavior, the CV curves show two pairs of redox peaks at 0.48/0.67 and 0.85/0.92 V. Compared with NVPF, the redox peaks of NVNPF x% shift to the low voltage, indicating that Ni element successfully replaced part of V element. Additionally, **Figure** **2**a displays the galvanostatic charge‐discharge (GCD) data of all samples (0.5 C), the obvious voltage platforms echo with the redox peaks in the CV results. The specific capacities of NVPF, NVNPF, NVNPF 5%, and NVNPF 10% are 35.53, 190.10, 158.34, and 141.27 mAh g^−1^, respectively. Among them, NVNPF exhibits the largest capacity, while the capacity decrease of NVNPF 20% (121.13 mAh g^−1^) is caused by excessive doping of Ni ions, indicating that the optimal doping ratio of Ni ions is 2.5%. Figure S9b (Supporting Information) shows the electrochemical impedance spectroscopy (EIS) plots of all samples with 1 Hz‐1 MHz frequency, and the semicircle in the high‐frequency region represents the charge transfer resistance, while the sloped line in the low‐frequency region denotes solid‐state diffusion of Na^+^. The Ni^2+^‐doping can enhance the conductivity of materials, introduce vacancy defects, and accelerate reaction kinetics. However, after charging/discharging 100 times in 17 m NaClO_4_‐EG electrolyte, the discharge capacity of NVNPF retains 53% of its initial capacity (Figure S9e, Supporting Information). It still faces challenges in preparing electrode materials with long cycle life to obtain high‐performance ARSIBs.

To further enhance the structural stability, NVNPF is combined with NC and etched by KOH to obtain NVNPF/NCK n% composites with the coexistence of crystalline and amorphous phases. In Figure S9i (Supporting Information), the CV results of NVNPF and NVNPF/NCK 5% exhibit two pairs of distinct redox peaks corresponding to V^3+^/V^4+^ redox reaction, observed at 0.48/0.67 and 0.85/0.92 V, respectively. With the increase of NC and KOH, there are three pairs of redox peaks in NVNPF/NCK at 0.3/0.45, 0.51/0.7, and 0.83/0.95 V, relating to V^3+^/V^4+^ redox reaction and the Na^+^ insertion/extraction behavior. The loading of NC increases the surface defects of the materials, shortens the ion transport path, and provides active sites, facilitating the Na^+^ transfer process. Figure 2b offers the GCD curves of the samples, the discharge capacities of NVNPF, NVNPF/NCK 5%, NVNPF/NCK, and NVNPF/NCK 20% are 190.10, 176.36, 187.26, and 126.10 mAh g^−1^, respectively. Moreover, NVNPF/NCK 20% exhibits the lowest discharge capacity due to excessive NC and KOH introduction, which greatly reduces the crystallinity of materials and leads to structural instability, resulting in side reactions and irreversible sodium loss during the charging/discharging process. As shown in Figure 2c, the EIS curves show that the charge transfer resistance follows the order: NVNPF/NCK < NVNPF < NVNPF/NCK 5% < NVNPF/NCK 20%, consistent with the GCD results. After charging/discharging 500 times at 1 C, the capacity retentions of NVNPF/NCK, NVNPF/NCK 5%, and NVNPF/NCK 20% are 58%, 81%, and 66%, respectively, with coulombic efficiencies (CE) of 97%, 97%, and 98% (Figure 2d), indicating that the optimal ratio of NC and KOH is 10%.

In Figure 2e, there are three distinct voltage platforms in the GCD data of NVNPF/NCK, which correspond to the CV results, and the discharge capacity of NVNPF/NCK is as high as 187.26 mAh g^−1^ (0‐1.4 V) at 0.5 C. Furthermore, Figure 2f presents the rate capability of NVNPF/NCK, with the current rate increasing to 2.5 C, the discharge capacity shows a decreasing trend. The specific capacity recovers to 94% of the initial capacity (175.28 mAh g^−1^) after restoring to 0.5 C, indicating the excellent stability and reversibility of NVNPF/NCK. As shown in Figure 2g, after charging/discharging 100 times (1 C), the capacity retention can be maintained at 53%, with a CE of 92%. In contrast, NVNPF/NCK exhibits higher capacity and more excellent cycling stability than NVNPF, the discharge capacity of NVNPF/NCK composites still maintains 81% of its initial capacity and a high CE of 97% after charging/discharging 500 times. Compared to previously reported cathode materials Na_3_V_2_(PO_4_)_2_F/C (NVPF/C),^[^ ^13^ ^]^ Na_2.2_V_1.2_Ti_0.8_(PO_4_)_3_ (NVTP),^[^ ^44^ ^]^ and Na_3_V_2_O_2x_(PO_4_)_2_F_3‐2x_/MWCNT (NVOPF),^[^ ^45^ ^]^ NVNPF/NCK shows superior electrochemical performance, making it a promising cathode material for ARSIBs (Figure 2h; Table S2, Supporting Information). On the one hand, replacing the V element with the Ni element improves the conductivity and structural stability of the materials. On the other hand, the introduction of NC and KOH increases the SSA and surface defects. The abundant defects and stable bonding in both crystalline and amorphous structures can reduce the volume expansion caused by the Na^+^ diffusion process, and maintain structural stability, so NVNPF/NCK composites with both crystalline and amorphous phases exhibit excellent cycling stability.

### 2.3 Na^+^ Storage Kinetics of NVNPF/NCK Cathode

The CV results are tested to further evaluate the Na^+^ storage kinetics of the NVNPF/NCK cathode. As the scan rate increased, the CV curves maintained a consistent shape, and the redox peak currents gradually increased, indicating that voltage polarization had little effect on the cathode. The peak currents *i* and scan rates ν of the cathode materials satisfy the equation:

(1) $$i=a\nu^{b}$$

**Figure 3:**
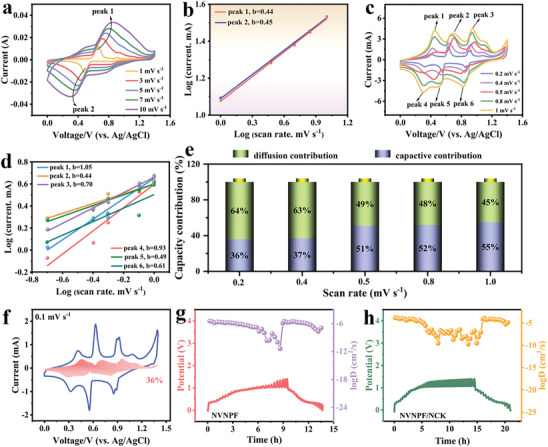
a) The CV curves and b) log (*i*) versus log (ν) curves of NVNPF. c) The CV curves and d) log (*i*) versus log (ν) curves of NVNPF/NCK. e) Capacitive and diffusion contributions and f) CV curves of NVNPF/NCK. The Na^+^ diffusion coefficients of g) NVNPF and h) NVNPF/NCK.

The correction formula is as follows:

(2) log(i)=log(a)+blog(ν)

in which, a and b are constants, and the b value is quantitatively evaluated by fitting*i*andνthrough Equation (2). Notably, the slopes of the fitting curves (b‐value) close to 0.5 or 1 indicate the diffusion‐controlled process or capacitive‐controlled process.^[^ ^46^ ^]^ In **Figure** **3**a, the CV curves of NVNPF are tested at different scan rates, the b values of NVNPF are 0.44 (peak 1) and 0.45 (peak 2), respectively (Figure 3b), demonstrating that the Na^+^ storage process of NVNPF is primarily diffusion‐controlled. Moreover, Figure 3c displays the CV results of NVNPF/NCK, the b values for the redox peaks 1–6 of NVNPF/NCK are 1.05, 0.44, 0.70, 0.93, 0.49, and 0.61, respectively (Figure 3d). These results indicate that the Na^+^ storage process in NVNPF/NCK is controlled by diffusion and capacitive process, and NVNPF/NCK is a battery‐supercapacitor composite material.

The contribution ratios of the diffusion and the capacitive controlled behaviors for the NVNPF/NCK can be calculated by the following equations:

(3) $$i=k_{1}\nu +k_{2}\nu^{1/2}$$

which can be simplified to

(4) $$i/\nu^{1/2}=k_{1}\nu^{1/2}+k_{2}$$

where *k*_1_ and *k*_2_ account for the constants. As shown in Figure 3e, the contributions of the diffusion‐controlled process are 64%, 63%, 49%, 48%, and 45%, and the contribution of the capacitive‐controlled process are 36%, 37%, 51%, 52%, and 55% at scan rates of 0.1, 0.2, 0.5, 0.8, and 1.0 mV s^−1^. Figure 3f shows that the capacitive contribution ratio accounts for 36% of the total capacitance at 0.1 mV s^−1^, demonstrating the diffusion‐controlled process of the energy storage mechanism at low scan rates. However, the insertion/extraction behavior of Na^+^ accelerates with the increase of scanning rates, which results in the limitation of the diffusion‐controlled process, and the capacitive contribution ratio gradually increases.^[^ ^47^ ^]^ Therefore, NVNPF/NCK exhibits excellent specific capacity and appealing cycling stability in aqueous electrolytes. The fabrication of battery‐supercapacitor composite material NVNPF/NCK with the coexistence of crystalline and amorphous phases, effectively addresses the structural instability issues of vanadium‐based polyanionic compounds in aqueous electrolytes.

The diffusion kinetics of NVNPF and NVNPF/NCK are further analyzed using the galvanostatic intermittent titration technique. The following formula can be used to evaluate the Na^+^ diffusion coefficient (*D_Na_*):

(5) $$D_{Na}=\frac{4}{\pi \tau }{\left(\frac{nV_{m}}{S}\right)}^{2}{\left(\frac{\Delta E_{s}}{\Delta E_{\tau }}\right)}^{2}$$

in which, τ is the pulse duration; *n* is the molar quantity; *V_m_* represents the molar volume; *S* is the electrode area; Δ*E_s_* is the open‐circuit potential difference, and Δ*E*_τ_ is the potential change during constant current pulses. The *D_Na_* of NVNPF/NCK is higher than that of NVNPF throughout the charging/discharging process at 0–1.4 V, demonstrating fast ion diffusion kinetics in NVNPF/NCK (Figure 3g,h). The battery‐supercapacitor composite materials NVNPF/NCK with the coexistence of crystalline and amorphous phases, are fabricated by loading NC onto NVNPF and etching with KOH, which greatly enhances the structural stability of materials and accelerates the Na^+^ diffusion kinetics.

### 2.4 Aqueous Screen‐Printed Inks

**Figure 4:**
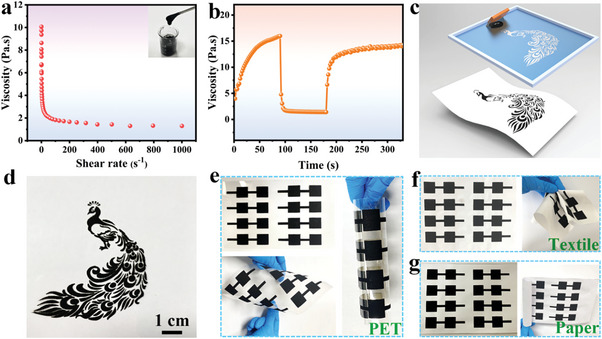
a) The relationship of viscosity and shear rates, b) viscosity versus shear rate of NVNPF/NCK‐based inks. c) The schematic illustration of the screen‐printing technique. d) The photographs of electrodes with complex patterns. Integrated flexible electrodes on flexible substrates such as e) PET, f) textile, and g) A4 paper.

The high‐performance NVNPF/NCK material is made into aqueous inks that exhibit typical thixotropic behavior, ensuring the integrity and accuracy of screen‐printed flexible electrode patterns, and maximizing performance efficiency. As shown in **Figure** **4**a, the viscosity of NVNPF/NCK‐based inks drops dramatically at shear rates between 0.1 and 200 s^−1^, then stabilizing below 2 Pa·s within the 100–1000 s^−1^ range, indicating its suitability for precise electrode patterning. The inserted photo demonstrates the smooth and viscousness of the NVNPF/NCK‐based inks. Moreover, Figure 4b presents the viscosity‐time curve, where the ink viscosity sharply drops upon the shear rates increase to 200 s^−1^, subsequently stabilizing ≈at 1.5 Pa·s. After the shear rate decreases to 0.1 s^−1^, the viscosity gradually returns to the initial level, which ensures the inks successfully pass through the screen to print the uniform and clear patterns, showing the highly elastic rheological properties of the inks. The screen‐printing technique is facile, scalable, and cost‐effective, enabling the production of fully‐printed flexible electrodes with various patterns, such as peacocks (Figure 4c,d), apples, doves, elephants, and the Chinese characters “elegant and graceful” (Figure S11a, Supporting Information). These fully‐printed electrodes exhibit excellent mechanical performance, maintaining clear and uniform patterns even after multiple bending cycles. To investigate the adhesion strength between the printed electrode and PET, the electrode is peeled off by a 3 M double‐sided adhesive tape. There is almost no film fracture and electrode material exfoliation, indicating outstanding adhesion (Figure S11b, Supporting Information). Moreover, the screen‐printing technique can realize the integration and high‐throughput manufacturing of flexible electrodes without connecting wires. The conductive inks are printed on flexible substrates like polyethylene terephthalate (PET) (Figure 4e), textile (Figure 4f), and A4 paper (Figure 4g) to obtain integrated flexible electrodes with clear patterns, and the size of square electrode is 2×2 cm^2^. Under various bending conditions, the printed electrodes on the three flexible substrates maintained the integrity and clarity of patterns. Even when bending inward and outward under external pressure, the flexible electrodes retained their original form, without damage, indicating the excellent flexibility and malleability of the printed electrodes (Figure S12, Supporting Information). Due to the numerous advantages, the screen‐printing technique is promising in the manufacturing of portable, wearable, and miniaturized flexible electronics.

### 2.5 Evaluation of Electrochemical Performance of F‐NTP//NVNPF/NCK Fully‐Printed ARSIBs

**Figure 5:**
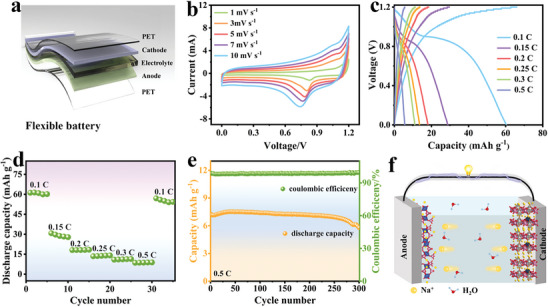
a) The schematic illustration of F‐NTP//NVNPF/NCK batteries. b) The CV curves, c) GCD results, d) rate capability, and e) cycle performance of the batteries. f) Working mechanism of this battery.

Inspired by the excellent electrochemical performance of NVNPF/NCK and F‐NTP electrode materials, the fully‐printed flexible ARSIBs (F‐NTP//NVNPF/NCK) with printed electrolyte are prepared by screen‐printing technique (**Figure** **5**a). To enhance the durability of the batteries, the assembled batteries are sealed in polyamides/polyethylene vacuum packaging bags by a vacuum heat‐sealing machine. Figure 5b shows the CV results of F‐NTP//NVNPF/NCK (0–1.2 V), which can maintain a similar shape at various scan rates, indicating outstanding stability. In Figure S13a(Supporting Information), the EIS curves are tested with 1 Hz–1 MHz frequency, and the insert photograph is packaged with flexible ARSIBs with a size of 2.2×2.2 cm^2^. To demonstrate excellent electrochemical performance, the GCD curves of F‐NTP//NVNPF/NCK are measured at different current rates (Figure 5c), and the obvious voltage platforms are attributed to the redox reactions in F‐NTP and NVNPF/NCK. As expected, the flexible batteries exhibit phenomenal electrochemical performance, with a discharge capacity of 60.37 mAh g^−1^ at 0.1 C, an energy density of 72.44 Wh kg^−1^, and a power density of 153.58 W kg^−1^ (based on the total mass of cathode and anode). Figure 5d displays the rate capability of the flexible battery, the capacity decreased at current rates of 0.1–0.5 C, while the capacity retention still maintains 93% after the current returns to 0.1 C, demonstrating outstanding reversibility and high stability. Additionally, the fully‐printed battery shows appealing cycling stability, the capacity retention is 80% and the CE is 99% after charging/discharging 300 times at 0.5 C (Figure 5e), further indicating the rapid diffusion kinetics and high stability of NVNPF/NCK and F‐NTP materials. The working mechanism of F‐NTP//NVNPF/NCK is the same as the typical rocking‐chair batteries. As shown in Figure 5f, Na^+^ is inserted/extracted between the cathode and anode through the electrolyte, accompanied by redox reactions, while electrons are transferred through the external circuit to power electronics.

**Figure 6:**
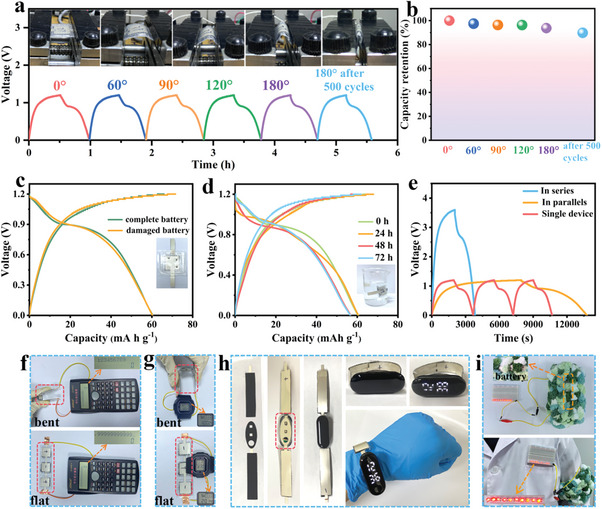
a) The charge/discharge curves and b) capacity retention of the batteries in bending states at 0.1 C. The GCD results of c) the punctured/complete batteries (the inset is a photograph of the damaged battery), d) the batteries are immersed in water for 24, 48, and 72 h, and e) a single battery and integrated batteries at 0.1 C. The integrated batteries can power f) calculators, and g) electronic watches in flat and bending states. h) The integrated energy wristband of the smart bracelet. i) The integrated batteries are woven into textiles to light up LEDs.

Although flexible ARSIBs show great promise for the development of portable and wearable electronics, they inevitably face various external damages in practical applications, such as bending, puncturing, and cutting. Therefore, it is important to ensure the safety and damage resistance of flexible ARSIBs for practical applications. The flexible thin electrodes were fabricated by printing the aqueous inks with highly elastic rheological properties onto PET substrates, the thickness of the electrode is ≈0.23 mm (including PET substrate and Ag current collectors and printed electrode materials). The thin electrode thickness and the strong adhesion between electrode material and PET substrate, enable the fully‐printed batteries with outstanding mechanical performance. The electrochemical performance of the F‐NTP//NVNPF/NCK batteries is tested at various bending angles of 0°, 60°, 90°, 120°, and 180° (**Figure** **6**a), the corresponding bending diameters of the bending states are 3, 2.6, 2.1, 1.5, and 0 cm (Figure S13b, Supporting Information), and the battery capacity retentions of the batteries under different bending states are 100%, 97%, 96%, 96%, and 94%, respectively. Notably, even after bending 500 times at 180°, the capacity retention of the battery still maintains 90% (Figure 6b). This is attributed to the loose contact between gel‐electrolyte and electrodes after repeated bending, which increases the ion diffusion distance and results in a slight decrease in battery capacity. In future studies, more advanced packaging technologies could be used to address this issue. The above results demonstrate that the fully‐printed batteries exhibit phenomenal mechanical performance and excellent electrochemical performance.

To further investigate the safety of these fully‐printed batteries, the electrochemical performance of the damaged battery is tested after the batteries are punctured with three holes using a 1.5 cm long, 0.1 cm diameter nail. Compared to the complete battery, the CV curves of the damaged battery show similar shapes and areas (Figure S14a, Supporting Information), indicating the high stability of these flexible batteries. In Figure 6c, the capacity of the damaged battery shows a little decrease (60.31 mAh g^−1^ at 0.1 C). In practical applications, it is necessary to ensure the waterproof and extreme temperature tolerance of flexible batteries to withstand extreme environments. As shown in Figure 6d, after the batteries are immersed in water for 24, 48, and 72 h, the discharge capacities are 60.28, 56.76, and 56.62 mAh g^−1^ respectively, with little decrease, and the results correspond to the CV curves (Figure S14b, Supporting Information). The appropriate packaging technology not only provides great waterproofing but also enhances the durability of the batteries. Figure S14c (Supporting Information) shows GCD curves (0.15 C) tested at −20, 25, and 40 °C to explore the potential applications of these batteries over a wide temperature range. The addition of EG co‐solvent endows the electrolyte to a low freezing point, the discharge capacity is 28.8 mAh g^−1^ at −20 °C, representing a 15% decrease compared to that (34.1 mAh g^−1^) at room temperature. The discharge capacity is 82.24 mAh g^−1^ at 40 °C, as the high temperature improves the ion conductivity of the electrolyte and accelerates ionic transfer in the gel‐electrolyte.^[^ ^48^ ^]^ The above indicates that the fully‐printed batteries possess excellent electrochemical performance, phenomenal mechanical performance, and remarkable safety.

Due to the superiorities of the facile procedure, high cost‐effectiveness, and scalability of the screen‐printing technique, the screen‐printed batteries can achieve patterning, miniaturization, and integration, which can design exclusive power supplies with different shapes to output the required voltage and capacity. As shown in Figure 6e, the GCD curves for a single battery and three batteries connected in series and parallel are tested at 0.1 C, which corresponds to the CV results (Figure S14d, Supporting Information). The output voltage of series‐connected batteries is 3.6 V, which is three times of the single battery (1.2 V), and the discharge capacity of parallel‐connected batteries is three times of the single battery, demonstrating exceptional performance uniformity. In addition, the series‐connected batteries can power electronics such as calculators (Figure 6f), electronic watches (Figure 6g), and timers (Figure S15, Supporting Information) for a long time in flat and bending states, indicating the significant potential of the integrated F‐NTP//NVNPF/NCK batteries. After the flexible batteries are cut multiple times (Figure S16, Supporting Information), the damaged batteries could still power the calculator, further demonstrating the high safety and damage resistance of the fully‐printed batteries. According to the design requirements of wearable electronics, the fully‐printed flexible batteries can be designed in various shapes and sizes in practical applications. As shown in Figure 6h, the integrated energy wristband can be manufactured using the screen‐printing technique, according to the internal structure of the smart bracelet. Three series‐connected flexible batteries are woven into textiles, which can successfully light up 10 LEDs (Figure 6i; Figure S17, Supporting Information), suggesting that flexible batteries are promising in the advancement of wearable electronics. The screen‐printed batteries with various planar geometries and complex patterns (apples, doves, elephants, and the Chinese characters “elegant and graceful”) are connected in series without metallic wires, which can power LEDs and an electronic watch in flat and bending states (Figure S18, Supporting Information). In summary, the F‐NTP//NVNPF/NCK fully‐printed batteries with the advantages of low cost, high flexibility, miniaturization, and integration are promising in practical applications.

## 3 Conclusion

The battery‐supercapacitor composite materials NVNPF/NCK with coexistence of crystalline and amorphous phases are prepared as the cathode for ARSIBs, indicating high capacity (187.26 mAh g^−1^), excellent energy density (262.16 Wh kg^−1^), and appealing cycling stability (500 cycles). In addition, the fully‐printed flexible ARSIBs F‐NTP//NVNPF/NCK are prepared using the screen‐printing technique, which presents a high discharge capacity of 60.37 mAh g^−1^, a phenomenal energy density of 72.44 Wh kg^−1^, and excellent cycling stability of 300 cycles. Even under extreme environments such as bending, puncturing, cutting, washing, and extreme temperatures, these batteries still maintain excellent electrochemical performance, phenomenal mechanical performance, and outstanding stability. Furthermore, the batteries can achieve tunability in thickness, size, and shapes, integration, and high‐throughput manufacturing to satisfy the design requirements of portable electronics. The integrated energy wristbands can be manufactured according to the internal structure of the smart bracelet, and the flexible batteries can be woven into textiles to power portable electronics. More importantly, taking into the high‐performance electrode materials and the inexpensive screen‐printing technique, the fully‐printed flexible ARSIBs F‐NTP//NVNPF/NCK hold significant potential as energy storage systems for wearable, flexible, and miniaturized electronics.

## Conflict of Interest

The authors declare no conflict of interest.

## Supporting information



Supporting Information

Supplemental Movie 1

Supplemental Movie 2

## Data Availability

The data that support the findings of this study are available in the supplementary material of this article.
