# Supplementary material for: Designing Crystalline/Amorphous NVNPF/NCK Cathode Toward High‐Performance Fully‐Printed Flexible Aqueous Rechargeable Sodium‐Ion Batteries (ARSIBs)
Source: Adv Sci (Weinh). 2025 Feb 4;12(12):2416120. doi: 10.1002/advs.202416120 (PMC11948014; doi:10.1002/advs.202416120)
Supplement: Supplementary file 1 — Supporting Information [file ADVS-12-2416120-s002.docx]

**Supporting Information**

**Designing crystalline/amorphous NVNPF/NCK cathode toward high-performance fully-printed flexible aqueous rechargeable sodium-ion batteries (ARSIBs)**

Hehe Ren, Jing Liang*, Qun Liu, Yuanjie Wei, Wei Wu*

Laboratory of Printable Functional Materials and Printed Electronics, School of Physics and Technology, Wuhan University, Wuhan 430072, PR China

*Corresponding Authors:

[jingliang@whu.edu.cn](mailto:jingliang@whu.edu.cn) (J. Liang)

[weiwu@whu.edu.cn](mailto:weiwu@whu.edu.cn) (W. Wu)

**Experimental Section**

1. **Materials and Reagents**

Ammonium metavanadate (NH_4_VO_3_), N,N′-methylenebisacrylamide (C_7_H_10_N_2_O_2_), acrylamide (C_3_H_5_NO), ammonium persulfate (H_8_N_2_O_8_S_2_), sodium perchlorate (NaClO_4_), and titanium isopropoxide (C_12_H_28_O_4_Ti) were was purchased from Aladdin Chemistry Co., Ltd. Sodium fluoride (NaF), ammonium phosphate monobasic (NH_4_H_2_PO_4_), citric acid monohydrate (C_6_H_8_O_7_·H_2_O), nickel acetate tetrahydrate (Ni(CH_3_COO)_2_·4H_2_O), p-benzoquinone (C_6_H_4_O_2_), o-phenylenediamine (C_6_H_8_N_2_), potassium hydroxide (KOH), sodium acetate anhydrous (CH_3_COONa), iron nitrate nonahydrate (Fe(NO_3_)_3_·9H_2_O), ethanol (CH_3_CH_2_OH), N-methylpyrrolidone (C_5_H_9_NO), ethylene glycol (C_2_H_6_O_2_), and polyvinylidene fluoride (PVDF) were bought from Sinopharm Chemical Reagent Co., Ltd. Ag conductive paste, acetylene black, and polyacrylic latex (LA133) were utilized as the current collector, conductive agent and binder for fabrication of printed electrodes, respectively.

1. **Synthesis of Materials**
   1. ***Synthesis of*** ***NVNPF***

Firstly, the NaF (6 mmol, 0.2519 g), NH_4_VO_3_ (3.9 mmol, 0.4563 g), Ni(CH_3_COO)_2_·4H_2_O (0.1 mmol, 0.0249 g), NH_4_H_2_PO_4_ (4 mmol, 0.4601 g) and C_6_H_8_O_7_·H_2_O (4 mmol, 0.8406 g) were dissolved in 30 mL deionized (DI) water in a three-necked round-bottom flask with magnetic stirrer. After stirring for 0.5 h, the mixed solution transformed into transparent solution, and then heated to 80 °C and maintained 80 °C until the solvent was evaporated to form into a gel. Next, the gel was dried at 120 °C for 12 h in a vacuum oven to obtain precursor powders. Finally, the precursor powders were heated at 700 °C (5 °C min^-1^) for 6 h under the protection of Ar flow to obtain NVNPF sample. The synthesis process of NVNPF x% (x=0, 2.5, 5, 10, 20) is consistent with the above.

- 1. ***Synthesis of NC***

Typically, the p-benzoquinone (0.06 mol, 6.48 g) and o-phenylenediamine (0.02 mol, 2.16 g) were dissolved in in 2000 mL ethanol solution with stirring. The mixture was heated to 50 °C, and stirred for 2 h to synthesize a polymer precursor. Following that, the resultant precursor was isolated by centrifugation, washed by DI water and ethanol, dried at 80 °C for 12 h. The resultant precursor was mixed with KOH (1:1, w/w) by grinding, and then, the mixture was kept 750 °C (3 °C min^-1^) for 2 h under a N_2_ flow to obtain NC.

- 1. ***Synthesis of NVNPF/NCK***

Firstly, 0.02 g the prepared precursor and 0.02 g KOH was mixed in 5 mL DI water in a beaker, and then, the 0.2 g NVNPF was added into the above mixture. After stirred for 5 h, the mixture was dried at 100 °C for 12 h in an oven. Next, the mixture was evenly grounded in an agate mortar. Finally, the mixture was transferred to the tube furnace, kept 700 °C (3 °C min^-1^) for 2 h under the N_2_ atmosphere to get the resulting product NVNPF/NCK.

- 1. ***Synthesis of F-NTP***

Firstly, the NaC_2_H_3_O_2_ (9 mmol, 1.2247 g), Fe(NO_3_)_3_·9H_2_O (3 mmol, 1.2120 g), NH_4_H_2_PO_4_ (9 mmol, 1.0353 g) and C_6_H_8_O_7_·H_2_O (9 mmol, 1.8913 g) were dissolved in 30 mL DI water in a three-necked round-bottom flask with magnetic stirrer to form the solution Ⅰ. The C_12_H_28_O_4_Ti (3 mmol, 0.8527 g) was dispersed in 20 mL ethanol solution to form solution Ⅱ, and the solution Ⅱ slowly dropped into solution Ⅰ (1 mL min^-1^). After stirred for 0.5 h, the mixed solution was heated to 80 °C, and maintained 2 h under sealed conditions. And then, the mixture still maintained 80 °C under open conditions until the solvent was evaporated to form into a gel. Next, the gel was dried at 80 °C for 24 h in a vacuum oven to obtain precursor powders. Finally, the powders were transformed in a tube furnace, and heated at 750 °C (5 °C min^-1^) for 10 h under the protection of Ar flow to obtain the product F-NTP.

1. **Electrochemical Test of Electrodes**

The active materials, acetylene black, and polyvinylidene fluoride (PVDF) were uniformly mixed with a weight ratio of 75: 15: 10 in N-methylpyrrolidone (NMP) solvent. The above mixture was coated on Ti mesh (100 mesh) to fabricate working electrodes (1 cm×1 cm) with the load mass of 10 mg cm^-2^ and thickness of 0.23 mm. The electrochemical performance of electrodes, such as the cyclic voltammetry (CV), galvanostatic charge/discharge (GCD), and electrochemical impedance spectroscopy (EIS) were explored by using CHI660e electrochemical workstation with a three-electrode system (Pt counter electrode and Ag/AgCl reference electrode). The Na^+^ diffusion coefficient was calculated by galvanostatic intermittent titration technique (GITT), and the GITT of the NVNPF and NVNPF/NCK cathode were tested on the LAND CT2001A battery testing system at the voltage window of 0-1.4 V. The GITT was set with a current pulse duration of 5 min at 0.3 C and a further interval time of 10 min.

1. **Preparation of 17 m NaClO_4_-EG gel-electrolyte**

Initially, abundant NaClO_4_ was dissolved in DI water and ethylene glycol (EG) mixed solution (V_DI_:V_EG_ = 3:1) to prepare 17 m (mol kg^-1^) NaClO_4_ electrolyte. Then 1.25 g C_3_H_5_NO was dissolved in 4.5 g 17 m NaClO_4_-EG electrolyte solution, and stirred for 1 h to obtain a transparent solution. After that, 150 μL C_7_H_10_N_2_O_2_ (10 mg mL^-1^) and 250 μL H_8_N_2_O_8_S_2_ (5 mg mL^-1^) were added into the solution. The mixture was printed on a flexible electrode, and cured at 80 °C for 0.5 h to obtain the 17 m NaClO_4_-EG gel-electrolyte.

1. **Electrochemical Test of Battery**

The aqueous ink with a solid concentration of 39 wt% was fabricated by mixing active materials, acetylene black, and LA133 aqueous binder according to a ratio of 74: 13: 13. The Ag paste was printed on PET to prepare the flexible current collector, and the aqueous ink was printed on the surface of Ag current collector to fabricated the flexible electrode (2×2 cm^2^) by using screen-printing technique. The thickness of the electrode is about 0.23 mm, including PET substrate and Ag current collectors (0.16 mm) and printed electrode materials (0.07 mm). The mass loading of the flexible electrodes was about 15 mg cm^-2^, and the mass ratio of the cathode and anode is 1:1.2.

The aqueous ink and screen-printing technique were used to fabricated the fully-printed ARSIBs with NVNPF/NCK cathode and F-NTP anode, and 17 m NaClO_4_-EG gel-electrolyte was printed on the surface of flexible electrode. In order to obtain more durable batteries, the prepared batteries were placed in polyamides/polyethylene (PA/PE) vacuum packaging bag, and a vacuum heat-sealing machine was used to fixed and sealed the batteries. The thickness of the flexible batteries is about 2.0 mm, and the length and width of these batteries are both 2.2 cm. The electrochemical performance and cycling stability of the batteries were tested by CHI 660E workstation with a three-electrode system and the LAND CT2001A, respectively. The energy density computation was based on the mass of cathode and anode, the computed formula is as follows:

$$Energy\text{ density=}\frac{\text{Discharge energy}}{Mass\text{ of cathode and anode}}$$

1. **Material Characterization**

The microstructure of active materials was observed by scanning electron microscopy (SEM, Tescan VEGA Compact) and energy dispersive spectroscopy (EDS, Tescan VEGA Compact). The structure of the samples was investigated by the X-ray diffraction instrument (XRD, [Bruker D8 Advance](http://gxxt.henu.edu.cn/Equipment/javascript:;" \o "X射线粉末衍射仪 (Bruker D8 Advance))), the Fourier transform infrared spectroscopy (FTIR), and Raman spectrum (XploRA plus, HORIBA Jobin Yvon). The valence states of the elements on the sample surface were characterized by the X-ray photoelectron spectra (XPS, ThermoFisher ESCALAB Xi+), and the ratio of the elements were characterized by the inductively coupled plasma mass spectrometry (ICP-AES/MS, Aglient-5110 OES). The rheological properties of ink were measured using the rotational rheometer (ARES-G2, TA). The electrochemical performance (CV, GCD, EIS, and GITT) and cycling stability of the battery was tested by CHI 660E workstation and LAND CT2001A.


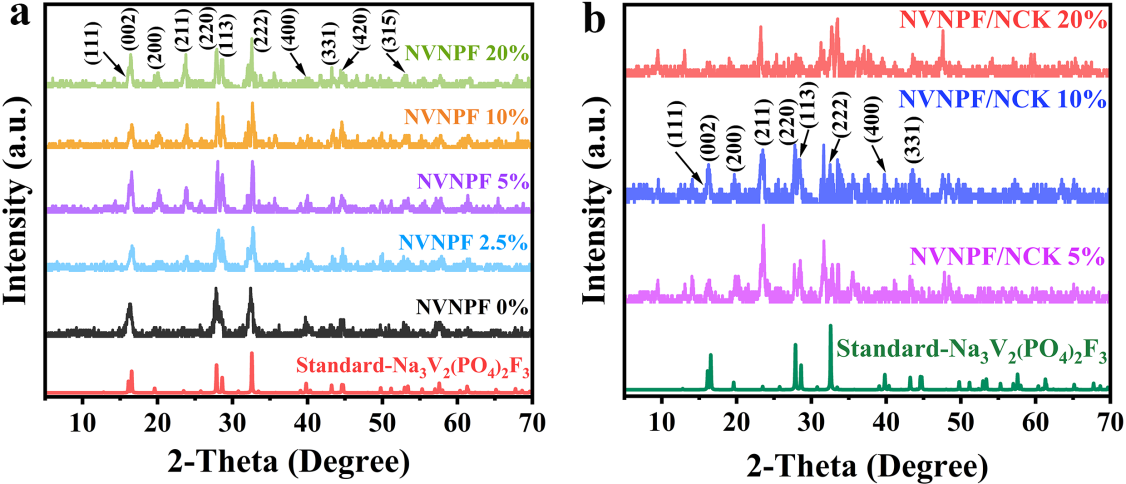


**Figure S1.** XRD patterns of a) NVNPF x% (x=0, 2.5, 5, 10, and 20), and b) NVNPF/NCK n% (n= 5, 10, and 20) samples.


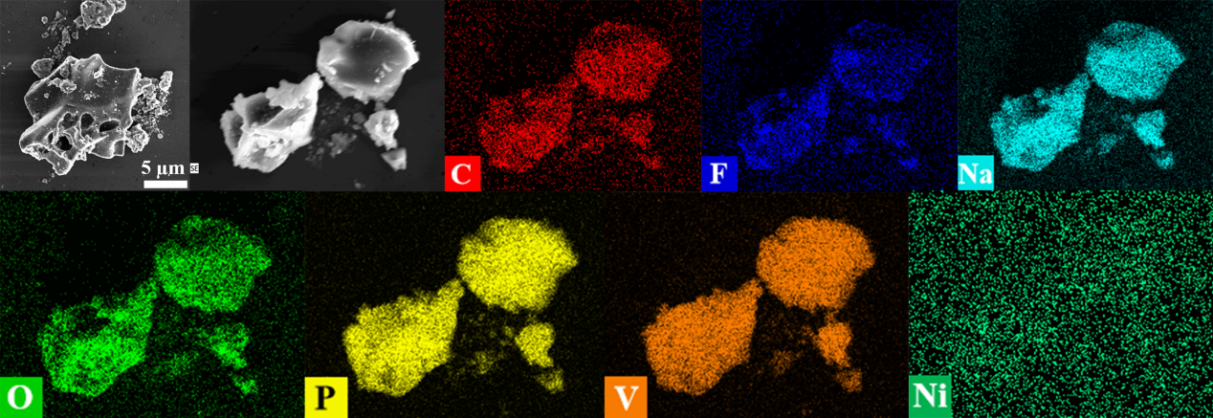


**Figure S2.** SEM and EDS mapping images of NVNPF samples.


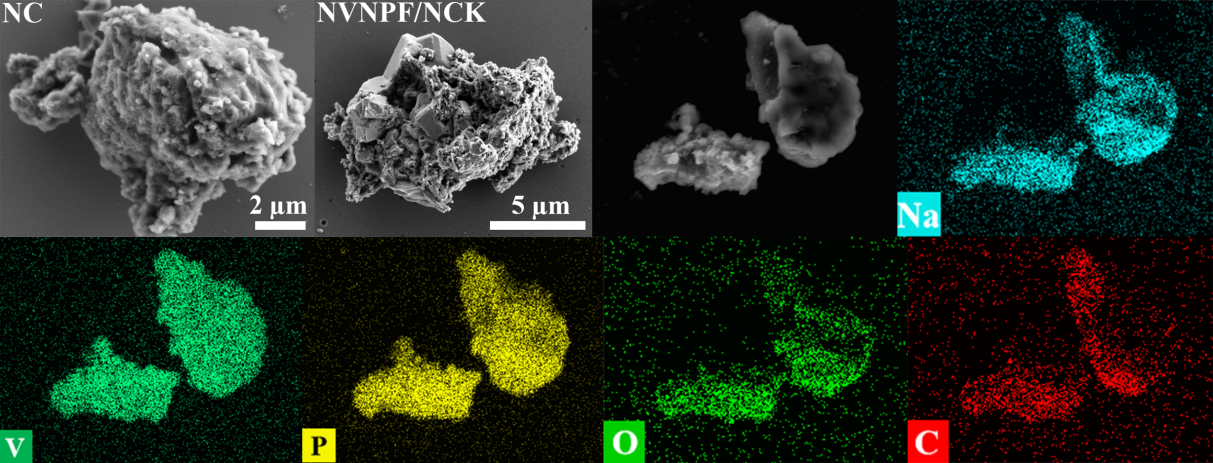


**Figure S3.** SEM and EDS mapping images of NVNPF/NCK samples.

As shown in **Figure S2**, the NVNPF with a size of approximately 10 micrometers (µm) exhibits irregular block-like structure. The elements C, F, Na, O, P, V, Ni are uniformly distributed in NVNPF, which demonstrates the successful doping of Ni element into NVPF materials. In **Figure S3**, the size of NVNPF/NCK is approximately 10-12 µm, and the surface is rough and porous, demonstrating that NC is successfully loaded on the NVNPF surface. Furthermore, C, F, Na, O, P, and V elements are uniformly distributed in NVNPF/NCK, although Ni element was not detectable due to its little content.


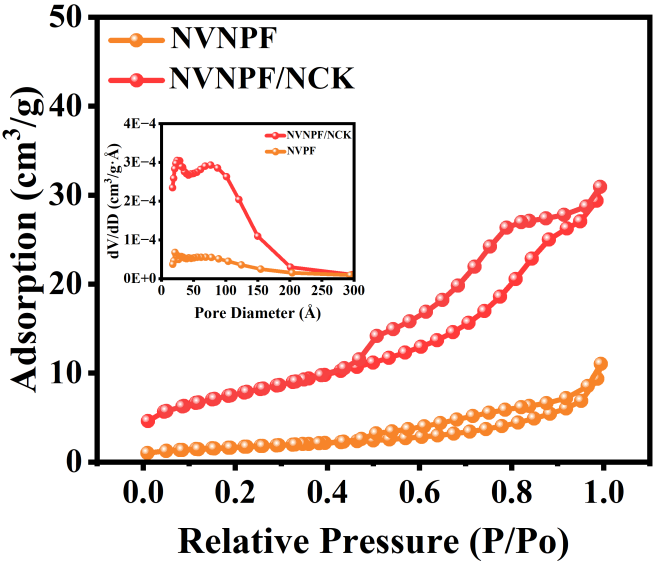


**Figure S4.** The specific surface area and pore size distribution of NVNPF and NVNPF/NCK.


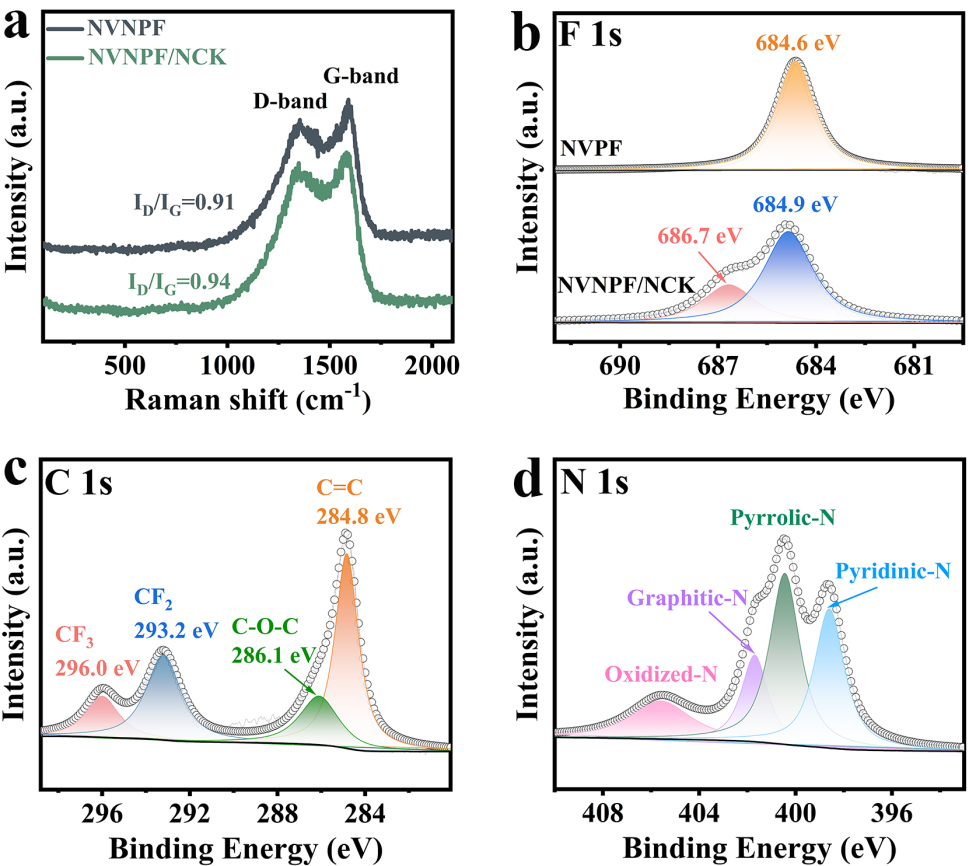


**Figure S5.** a) Raman spectrum of NVNPF and NVNPF/NCK samples. XPS spectrum: b) F 1s for NVPF and NVNPF/NCK, c) C 1s and d) N 1s for NVPF.

As shown in Figure S5c, the peaks at 284.8 and 286.1 eV in the C 1s spectrum correspond to the C=C and C-O-C bonds, while the binding energy at 284.8 and 286.1 eV is the signal of C-F_2_ and C-F_3_ bonds.^[1,2]^ In the N 1s spectrum (Figure S5d), the peaks at 398.6, 400.5, 401.7, and 405.6 eV are attributed to pyridinic-N, pyrrolic-N, graphitic-N, and oxidized-N, respectively,^[3]^ confirming the successful synthesis of NVNPF/NCK.

**Table S1.** The test results of inductively coupled plasma atomic emission spectroscopy (ICP-AES).

|  | sample | Element | Element content W (%) |
| --- | --- | --- | --- |
| 1 | F-NTP | Fe | 1.41% |
|  |  | Ti | 8.81% |


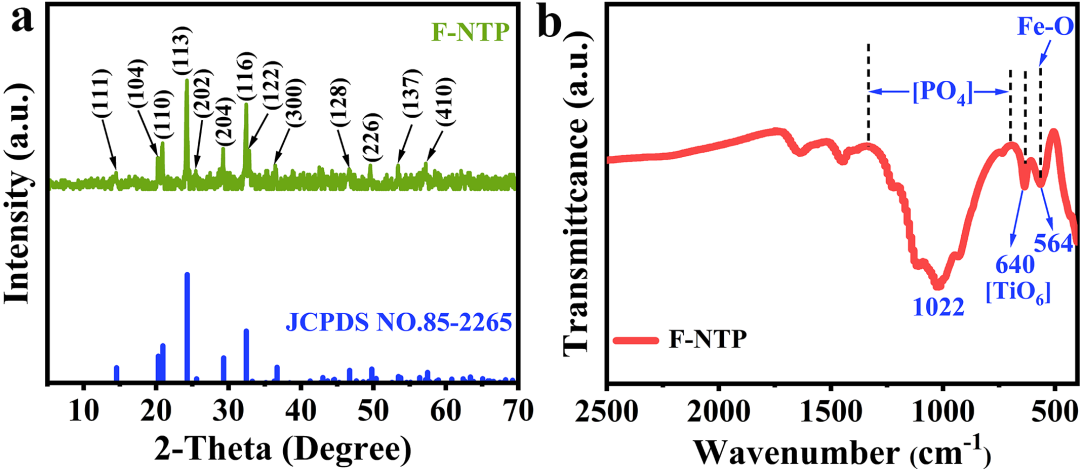


**Figure S6.** a) XRD pattern and b) FTIR spectrum of F-NTP samples.

The Na super ionic conductors (NASICON) materials NaTi_2_(PO_4_)_3_ (NTP) with 3D open framework structure are composed of TiO_6_ octahedra and PO_4_ tetrahedra.^[4-6]^ The unique structure provides enough space for rapid Na^+^ transfer process, making NTP a promising anode material for ARSIBs. However, the operating potential of NTP anode (-0.807 V vs. Ag/AgCl) is close to the hydrogen evolution potential of water (-0.817 V vs. Ag/AgCl), which results in hydrogen evolution reactions during the charging/discharging process, hindering the practical application of NTP as anode materials for ARSIBs.^[7]^ The F-NTP is fabricated by doping Fe^3+^ to replace Ti^3+^, and the redox reactions during charge-discharge are facilitated by the synergistic effect of Ti^4+/^Ti^3+^ and Fe^3+^/Fe^2+^ redox couples (**Table S1**). Moreover, the operating potentials improved to -0.778 V vs. Ag/AgCl, which prevent prevents overlap with the hydrogen evolution potential of water, effectively improve the stability of F-NTP in aqueous electrolyte. As shown in **Figure S6**a, the XRD pattern of F-NTP matches the characteristic peaks of the standard pattern for rhombohedral phase NTP (JCPDS No. 85-2265),^[8]^ indicating that the doping of Fe^3+^ has little effect on the crystal structure of NTP. In Figure S6b, the FTIR results are tested to further investigate the structure of F-NTP. The signal for Fe-O bonds is observed at 564 cm^-1^,^[9]^ and the signal at 640 cm^-1^ corresponds to the [TiO₆] units.^[10]^ Furthermore, the signal at 1022 cm^-1^ represents the asymmetric stretching vibrations of P-O bonds, which belong to the [PO₄] units.^[11]^ The FTIR results confirm the successful doping of Fe^3+^ and indicate without altering the [TiO₆] and [PO₄] units.


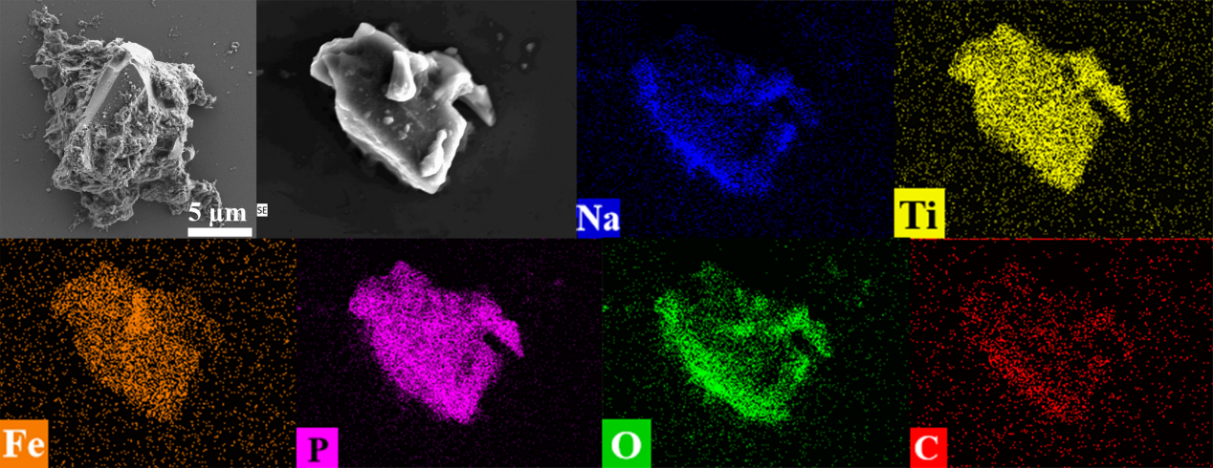


**Figure S7.** SEM and EDS mapping images of F-NTP samples.


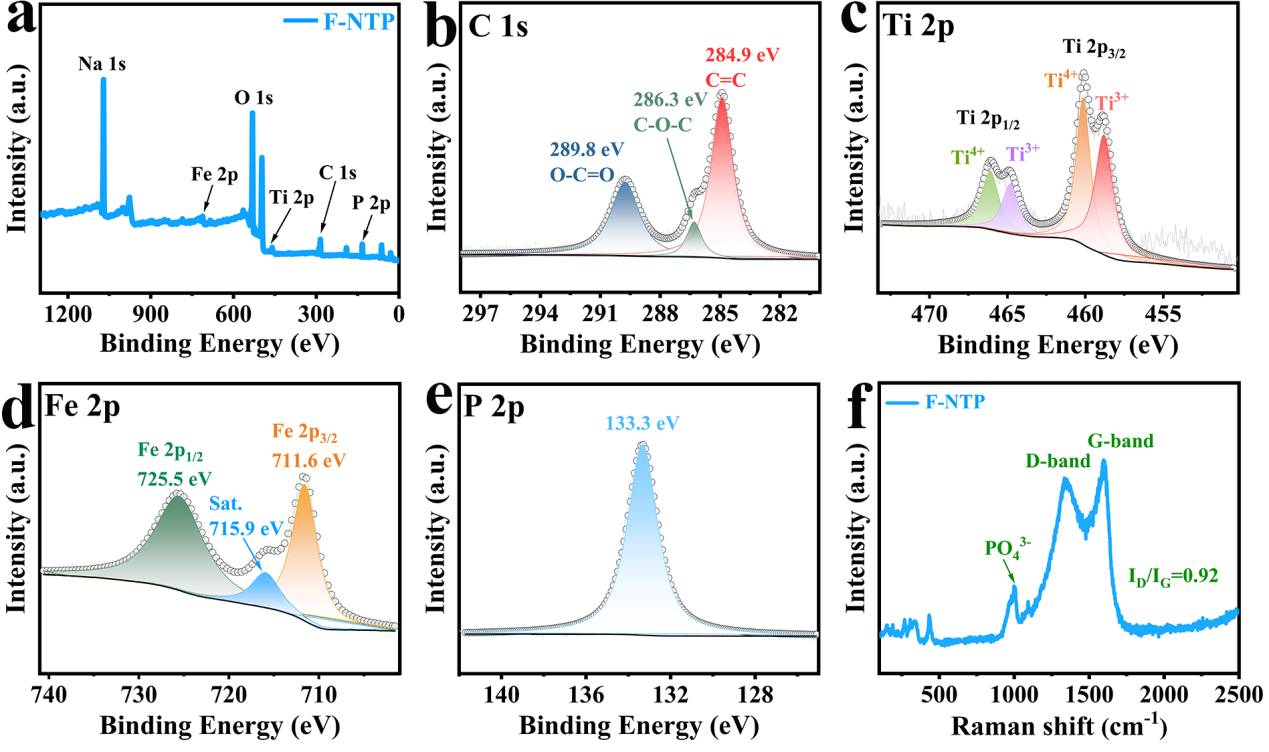


**Figure S8.** XPS spectrum of F-FNTP: a) full scan spectrum, b) C 1s, c) Ti 2p, d) Fe 2p, and e) P 2p. f) Raman spectrum of F-NTP.

As shown in **Figure S7**, the surface morphology and structural composition of the F-NTP are measured by the SEM. The surface morphology of F-NTP is irregular blocks with dimensions of approximately 10 µm, which exhibits abundant pores on the surface. Additionally, the Na, Ti, Fe, P, O, and C elements are uniformly distributed in the EDS images of F-NTP, and the C element are attributed to the carbon layer on the surface, further confirming the successful synthesis of F-NTP. In Figure S7, the detailed analysis of the composition and elemental valence states of F-NTP are tested by the XPS. As shown in **Figure S8**a, the XPS full spectrum displays the presence of Na, Fe, O, Ti, C, and P in the sample, indicating successful synthesis of the materials. In the C 1s spectrum (Figure S8b), the peaks at 284.9, 286.3, and 289.8 eV correspond to C=C, C-O-C, and O-C=C bonds, respectively.^[12]^ Moreover, the Ti 2p spectrum (Figure S8c) shows two peaks at 458.8 and 464.8 eV, which are associated with 2p₃/₂ and 2p₁/₂ states of Ti^3+^, and peaks at 460.1 and 466.1 eV correspond to 2p₃/₂ and 2p₁/₂ states of Ti⁴⁺.^[13]^ In the Fe 2p spectrum (Figure S8d), the signal peaks of Fe 2p₃/₂ and Fe 2p₁/₂ states are located at 711.6 and 725.5 eV, and a satellite peak are observed at 715.9 eV.^[14]^ The bonding energy at 133.3 eV are attributed to the signal P 2p in the [PO₄] unit (Figure S8e).^[15]^ The XPS results confirm that the successful doping of Fe^3+^ did not affect the valence states of other elements. In addition, the structural characteristics of F-NTP is further investigated by the Raman spectroscopy (Figure S8f). The signal peaks of D and G band is attributed to the carbon layer formed on the surface of F-NTP after high-temperature calcination.


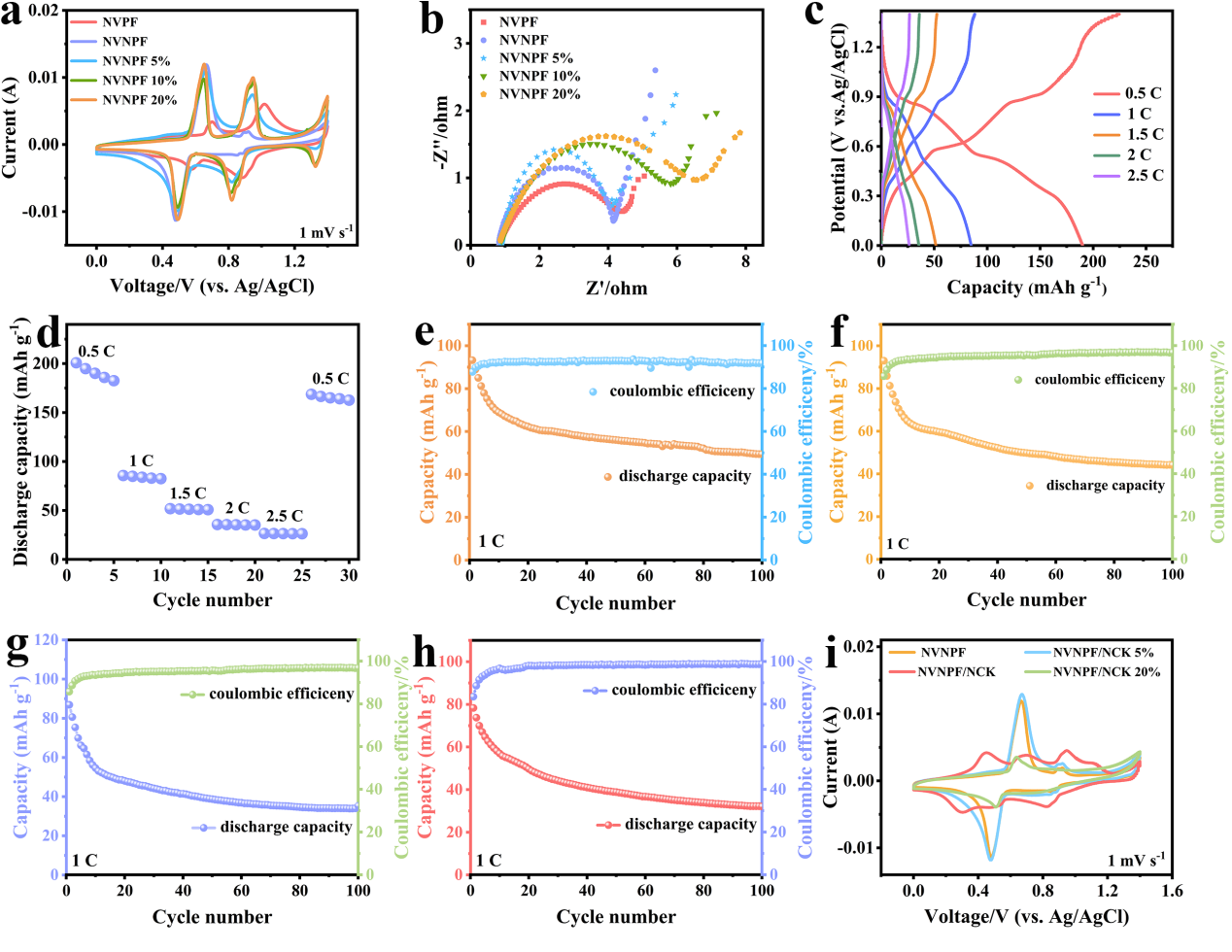


**Figure S9.** a) CV curves (1 mV s^-1^) and b) EIS data of NVNPF x% (x=0, 2.5, 5, 10, and 20). c) GCD curves and d) rate capability of NVNPF. The cycle performance of e) NVNPF, f) NVNPF 5%, g) NVNPF 10%, and h) NVNPF 20% at 1 C. i) CV curves of NVNPF/NCK n% (n= 0, 5, 10, and 20).

As shown in Figure S9c, the GCD curves of NVNPF are examined at the current rates of 0.5-2.5 C within the voltage range of 0-1.4 V. Two voltage platforms are obviously observed in GCD curves, corresponding to the two pairs of redox peaks of the CV curves. Figure S9d presents the rate performance at various current rates, the discharge capacity decreased with the current rate increased from 0.5 C to 2.5 C. While the current rates are reduced from 2.5 C back to 0.5 C, the discharge capacity recovered to 81% of its initial value, suggesting that the insertion/extraction behavior of Na⁺ is controlled by the ion migration rate. To further explore the effect of Ni^2+^-doping on the cycling stability, the cycle life of NVNPF x% materials is evaluated. After charging/discharging 100 times, the capacity retention rates of NVNPF, NVNPF 5%, NVNPF 10%, and NVNPF 20% are 53%, 48%, 41%, and 41% (at 1 C), respectively (Figure S9e-h). The substitution of some larger-radius V ions with smaller-radius Ni ions leads to lattice contraction, shortens the migration paths for Na⁺, improves the structural stability.


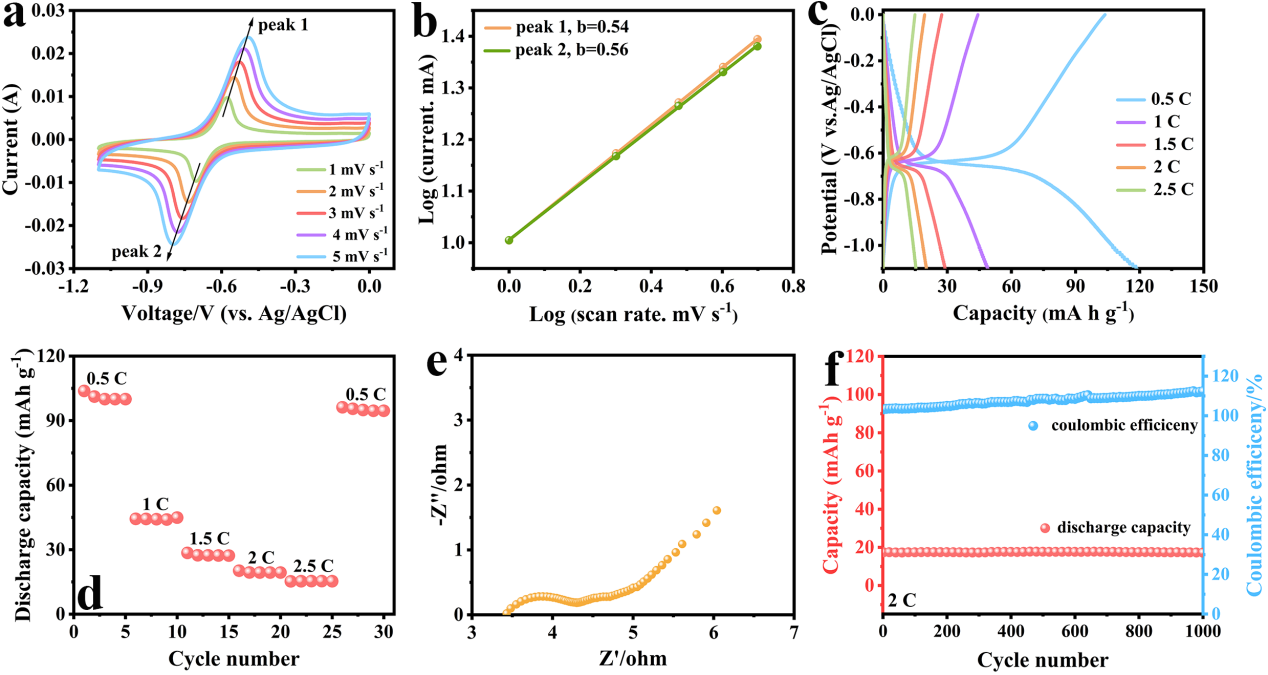


**Figure S10.** The electrochemical performance of F-NTP. a) CV curves at 1-5 mV s^-1^, b) the$\log(i)$versus$\log(\nu)$curves of F-NTP anode, c) GCD curves, d) rate capability, e) EIS data, f) cycling stability at 2 C.

To investigate the Na^+^ storage mechanism of F-NTP, the CV curves are tested at scan rates of 1, 2, 3, 4, and 5 mV s^-^¹ (**Figure S10**a). The peak current$i$and scan rate $\nu$are analyzed using the modified coincidence formula $\log(i)=\log(a)+b\log(\nu)$to fit the redox peaks, and the slope b values are 0.54 (peak 1) and 0.56 (peak 2), indicating that the Na^+^ storage process of F-NTP is controlled by diffusion processes (Figure S10b). As shown in Figure S10c, the F-NTP exhibits a high discharge capacity of 118.71 mAh g^-1^ at 0.5 C, and the GCD curves show obvious charging/discharging platforms at -0.58 and -0.7 V, which are consistent with the redox peaks observed in the CV curves. Additionally, the rate capability of the F-NTP anode is tested at various current densities (Figure S10d). The discharge capacity decreases with increasing current rates, while the current rate is reduced from 2.5 C back to 0.5 C, the discharge capacity can recover to 95% of the initial capacity, demonstrating excellent reversibility and stability of F-NTP. In Figure S10e, the EIS curve of F-NTP is tested at the frequency of 1 Hz-1 MHz, which is composed of high-frequency semicircle (charge transfer resistance) and low-frequency sloped line (Na^+^ solid-state diffusion). The F-NTP shows excellent cycling stability in 17 m NaClO_4_-EG electrolyte, even after 1000 cycles at a high current rate of 2 C, the discharge capacity still maintains 97% of the initial capacity, and Coulombic efficiency (CE) is approximately 100% (Figure S10f).

**Table S2.** The electrochemical performance of several cathode materials.

|  | Potential | Capacity (capacity/current density) | Energy density | Power density | Cycle performance  (capacity retention/cycles/CE) | Ref. |
| --- | --- | --- | --- | --- | --- | --- |
| NVPF/C | 1.4 V | 132.7 mAh g^-1^/0.5 C | 185.78 Wh kg^-1^ | 237.27 W kg^-1^ | 45%/100 cycles/98% | [13] |
| NVTP | 0.5 V | 62 mAh g^-1^/1 C | 31 Wh kg^-1^ | 64 W kg^-1^ | 93%/500 cycles/97% | [44] |
| NVOPF | 0.9 V | 65 mAh g^-1^/1 C | 58.5 Wh kg^-1^ | 115.2 W kg^-1^ | 71%/100 cycles/98% | [45] |
| NVNPF/NCK | 1.4 V | 187.26 mAh g^-1^/0.5 C | 262.16 Wh kg^-1^ | 179.46 W kg^-1^ | 81%/500 cycles/97% | This work |


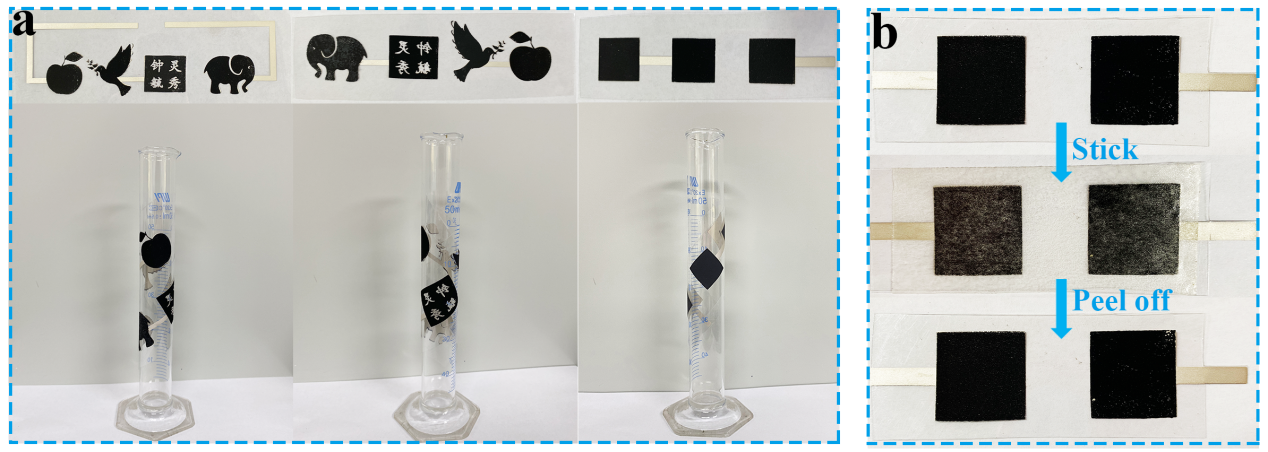


**Figure S11.** a) The photographs of electrodes with various patterns connected in series at bending state. b) The adhesion strength test of screen-printed electrodes.


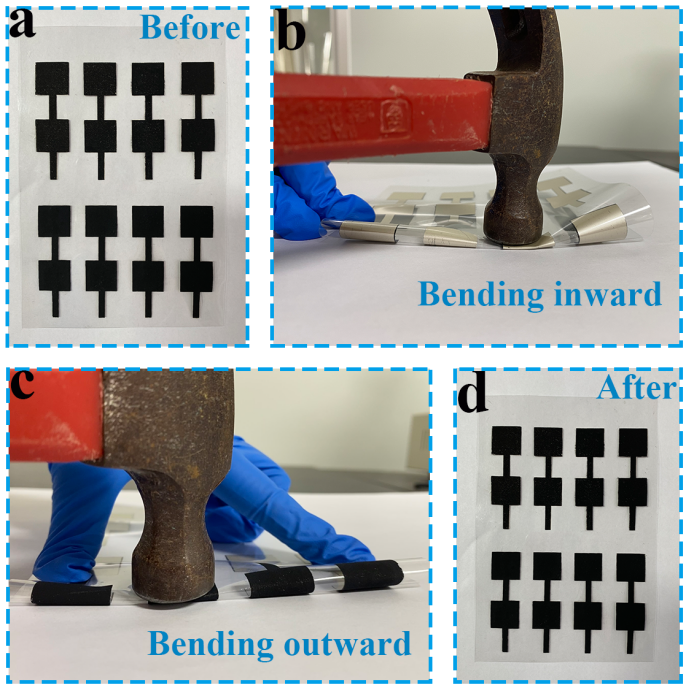


**Figure S12.** The photographs of electrodes a) before bending, b) bending inward, c) bending outward, and d) after bending.


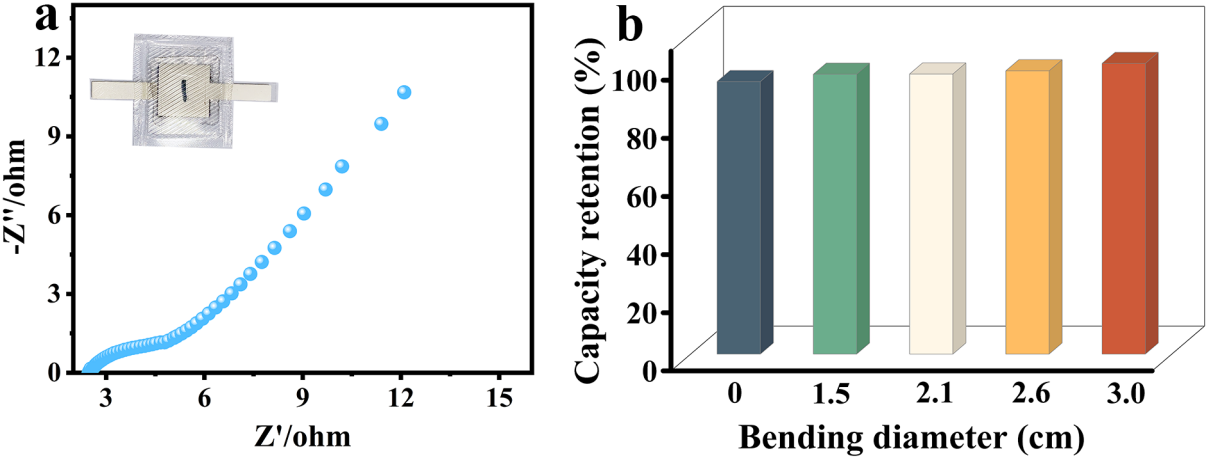


**Figure S13.** a) The EIS data of F-NTP//NVNPF/NCK batteries, the picture inserted is the photograph of the flexible battery. b) The variation of battery capacity with diverse bending diameters at 0.1 C.


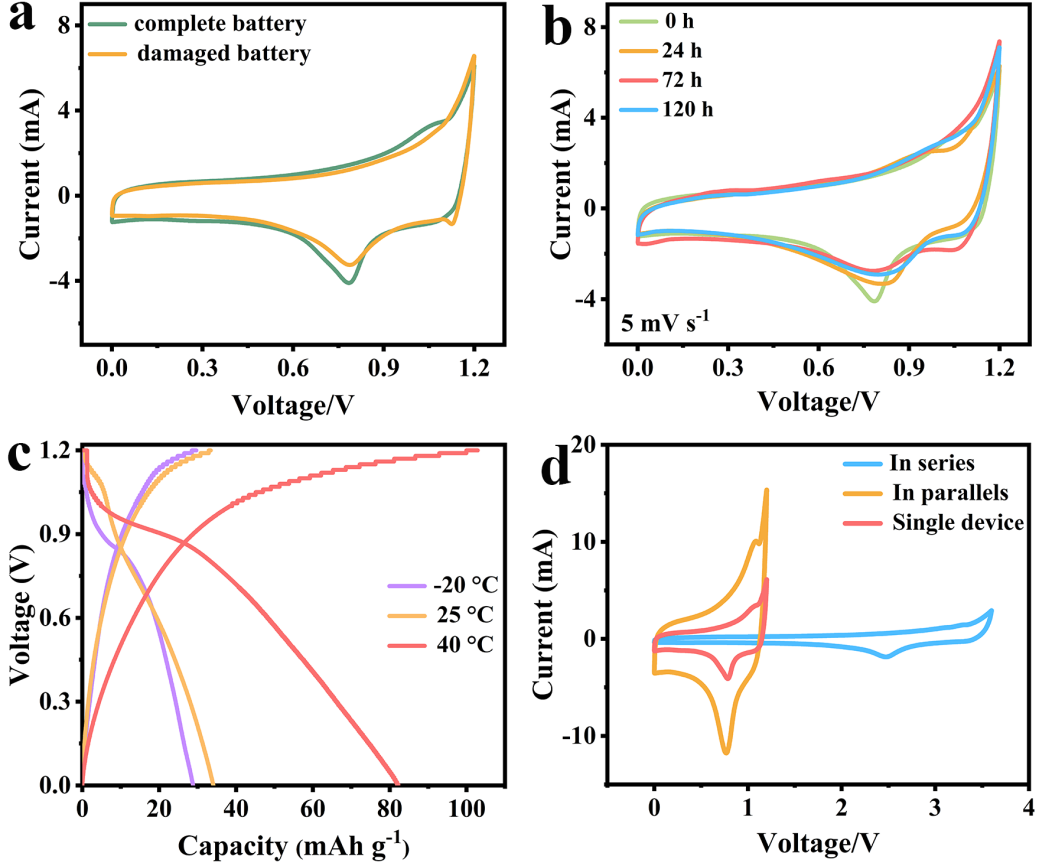


**Figure S14.** a) The CV curves of complete and damaged batteries. b) Waterproof performance of the flexible batteries. c) The discharge capacities of the batteries at -20 °C, 25 °C, and 40 °C. d) The CV curves of the signal battery, three flexible batteries connected in series and parallels.


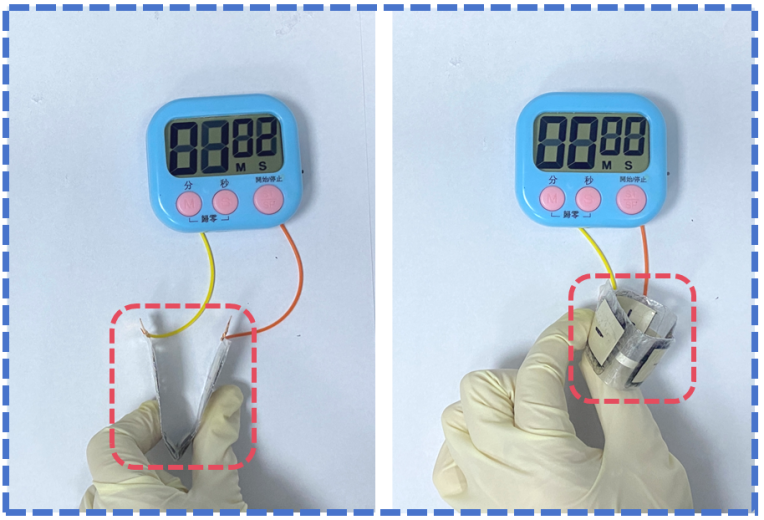


**Figure S15.** Three flexible batteries connected in series can power a timer in bending states.


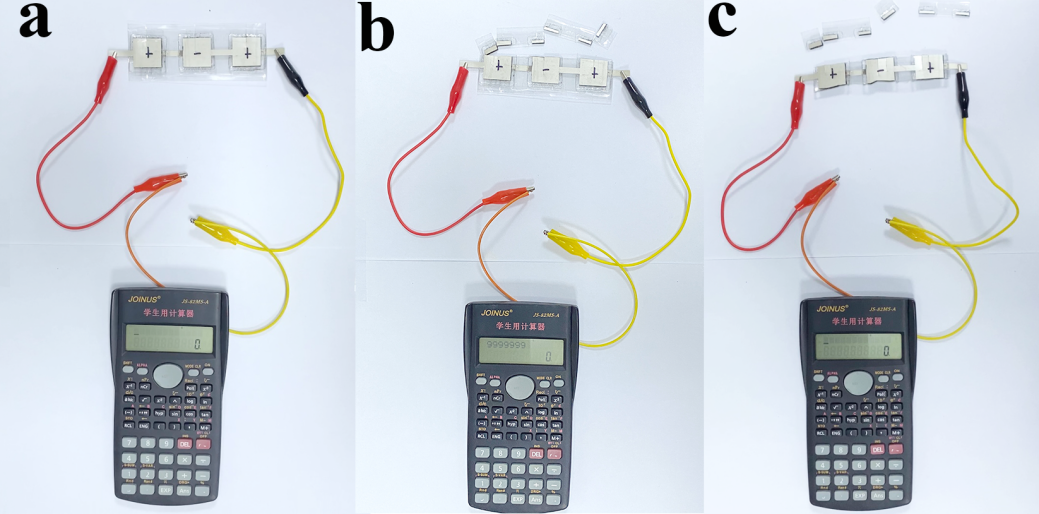


**Figure S16.** The damaged batteries can power the calculator even after being cut.


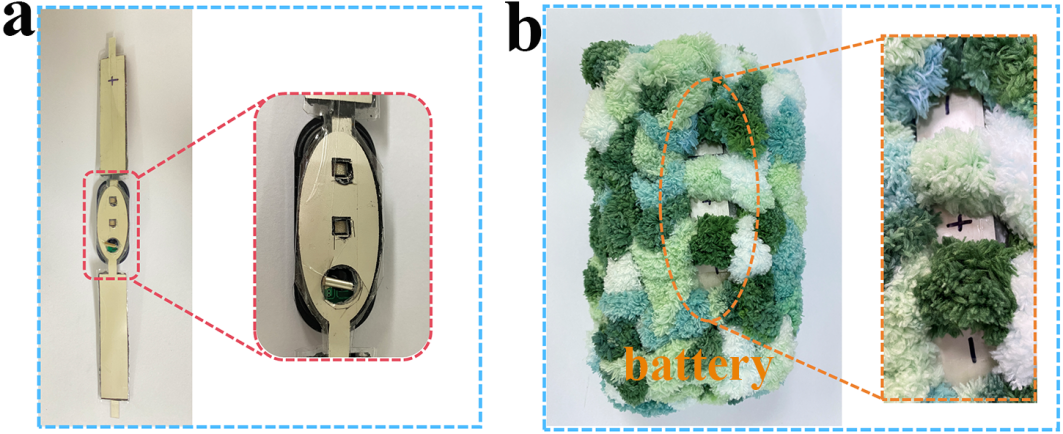


**Figure S17.** a) The batteries can be designed in various shapes according to the internal structure of the smart bracelet, b) the integrated flexible batteries are woven into textiles.


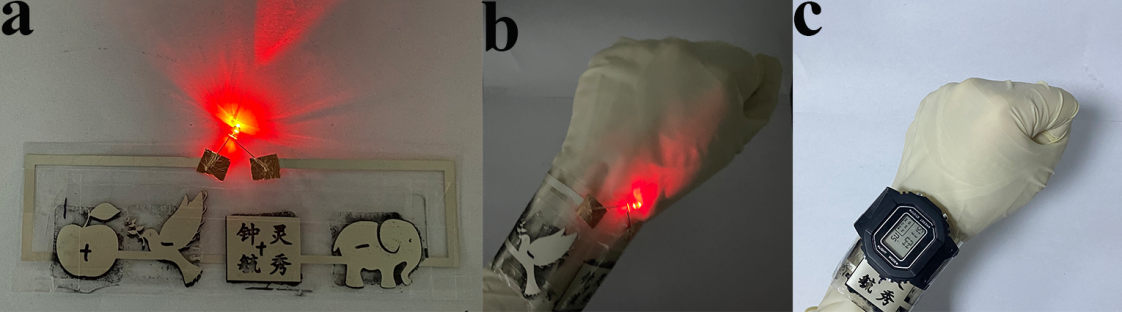


**Figure S18.** The fully-printed batteries with various shapes can light up LED and power an electronic watch in in flat and bending states.

**References**

[1] S. M, W. Liu, D. Zhang, C. Yang, Y. Luo, X. Lou, R. Guo, Y. Wang, J. Xie, *Chem. Eng. J* **2023**, *474*, 145819.

[2] Q. Meng, J. Shao, X. Dou, H. Chi, *Small* **2024**, *20*, 2308483.

[3] G. Gan, X. Li, L. Wang, S. Fan, J. Li, F. Liang, A. Chen, *ACS Catal*. **2019**, *9*, 10931-10939.

[4] M. G. Wu, W. Ni, J. Hu, J. M. Ma, *Nano-Micro Lett*. **2019**, *11*, 1-36.

[5] Z.-G. Liu, R. Du, X.-X. He, J.-C. Wang, Y. Qiao, L. Li, Chou, S.-L. Chou, *ChemSusChem* **2021**, *14*, 3724-3743.

[6] Q. Ni, Y. Bai, F. Wu, C. Wu, *Adv. Sci*. **2017**, *4*, 1600275.

[7] Y. Qiu, Y. Yu, J. Xu, Y. Liu, M. Y. Ou, S. Sun, P. Wei, Z. Deng, Y. Xu, C. Fang, Q. Li, J. Han, Y. Huang, *J. Mater. Chem. A* **2019**, *7*, 24953.

[8] Q. Deng, Q. Cheng, X. Liu, C. Chen, Q. Huang, J. Li, W. Zhong, Y. Li, J. Hu, H. Wang, L. Wu, C. Yang, *Chem. Eng. J*. **2019**, *430*, 132710.

[9] Y. Cao, Y. Liu, D. Zhao, J. Zhang, X. Xia, T. Chen, L. Zhang, P. Qin, Y. Xia, T. Chen, L.-C. Zhang, Y. Xia, *J. Alloys Compd*. **2019**, *784*, 939-946.

[10] G. Pang, P. Nie, C. Yuan, L. Shen, X. Zhang, H. Lia, C. Zhang, *J. Mater. Chem. A* **2014**, *2*, 20659.

[11] G. Pang, C. Yuan, P. Nie, J. Zhu, X. Zhang, H. Li, B. Ding, *Appl. Mater. Today* **2016**, *4*, 54-61.

[12] H.-K. Roh, H.-K. Kim, M.-S. Kim, D.-H. Kim, K. Y. Chung, K. C. Roh, K.-B. Kim, Nano Res. **2016**, *9*, 1844-1855.

[13] Z. Liu, Y. An, G. Pang, S. Dong, C. Xu, C. Mi, X. Zhang, *Chem. Eng. J*. **2018**, *353*, 814-823.

[14] Y. Cao, Y. Liu, D. Zhao, X. Xia, L. Zhang, J. Zhang, H. Yang, Y. Xia, *ACS Sustainable Chem. Eng*. **2020**, *8*, 1380-1387.

[15] S. Xu, H. Chen, X. Zhang, M. Zhou, H. Zhou, *ACS Appl. Mater. Interfaces* **2023**, *15*, 47764-47778.
